# Estimation of Underreported Cases of Infections and Deaths from COVID-19 for Countries with Limited and Scarce Data: Examples from Nepal

**DOI:** 10.1155/2022/3276583

**Published:** 2022-01-10

**Authors:** Jyoti U. Devkota

**Affiliations:** Department of Mathematics, Kathmandu University, Dhulikhel, Nepal

## Abstract

COVID-19 pandemic has overburdened the public healthcare system around the world. Further, lockdown imposed to curb the spread of pandemic has shown to have an adverse effect on economic and health status of an individual. It has also compelled us to switch from the physical world to virtual world, thus depriving us of benefits of person-to-person direct contact. People from developing countries are specially affected. An average person here lacks basic skills needed to survive in the digital world. Due to limited COVID-19 testing capacities in such countries, there is also less testing. Less testing means less contact tracing, underreported cases, and rapid spread of disease. In this paper, the underreported cases of daily infections and daily deaths are predicted using mathematical models. This is based on daily data published by the Government of Nepal. Here, Kathmandu valley is taken as a model area for estimation of underreporting. The behavior of probability of infection, probability of recovery, and probability of deaths is also mathematically analyzed. A time-dependent susceptible infected and recovered model is also proposed. Here, the second wave of COVID-19 is analyzed in detail from 1 Feb 2021 to 1 June 2021. The effect of lockdown on the psychology of people is also modeled with principal components analysis. The inherent and latent factors affecting the people in lockdown are identified. This is based on detailed primary data collected from a survey of 277 households.

## 1. Introduction

Countries in the developing world have poor health infrastructure. COVID-19 pandemic has put a lot of pressure on public healthcare system of such countries. This healthcare system is already understaffed and underresourced. PCR test is used for detecting COVID-19. It is also called swab test. Nepal has the capacity to conduct PCR tests on 23,000 samples on average daily. But tests are conducted only on 15000 samples on average daily [1]. The government of Nepal has announced free COVID-19 tests and treatment for its citizens. But the services are very fast and efficient in the private sector. PCR test costs around 20 USD in the private sector. This is beyond the reach of an average citizen of such countries. These tests are sometimes unreliable, as they give negative results during the first time and positive results during the second time. Thus, less people turn up for testing. This has resulted in less contact tracing and rapid increase in infections. Hence, there are underreported cases of infections and deaths from COVID-19.

In Nepal, the first case of COVID-19 was reported on 23 January 2020. It was a 32-year-old man who had recently returned from Huwei, China. The patient recovered and contacts were also asymptomatic [2]. The Government of Nepal enforced a strict lockdown from 24 March 2020. This lockdown was completely relaxed on 19 September 2020.

The second wave of COVID-19 epidemic in Nepal was triggered in April 2021. Second wave of COVID-19 epidemic in India was the cause. It was due to Nepalese migrant workers coming back to Nepal. This was after the announcement of lockdowns and curfews in different parts of India [3]. Nepal shares an open border with its southern neighbor India. People from either side of the border can freely cross and work without work permits [4]. Initially the positive cases of COVID-19 were mostly either Indian nationals working in Nepal or Nepali workers who had recently returned from India. Thousands of workers had returned to Nepal without proper screening. This was also the scenario in the first wave. Many workers were stranded in different parts of India due to lockdowns in both countries. Many workers were stranded in the border [5–9].

With the onset of second wave of COVID-19 infections, the government of Nepal enforced a strict lockdown on 29 April 2021. This lockdown was partially relaxed from 22 June 2021 [10]. From 22 June, vehicles could move on the road. This was according to the odd and even numbers of the number plate. The shops and departmental stores were allowed to be open till 11:00 a.m. only.

The capital city Kathmandu is still the hotspot of COVID-19 pandemic [11–13]. The Government of Nepal had started the COVID-19 vaccination drive in January 2021. Around 10% of the total population had received the first dose of COVID-19 vaccine by May 2021 [14, 15].

In this paper, the underreported cases of daily infections and deaths are estimated for Kathmandu valley. This is for the period 1 Feb to 1 June 2021. Daily COVID-19 updates of the Government of Nepal provide detailed information for Nepal. These data are used here. But in these updates, detailed information on individual areas including Kathmandu valley is missing. Kathmandu valley is the area with the highest population density in Nepal. Data on the daily testing, infections, deaths, and recovery for Nepal are used for drawing strength for this estimation and inference. As the data collection process of COVID-19 is being monitored globally by the World Health Organization [16], it is assumed that the pattern exhibited by these data for Nepal is correct but underreported. The behavior of these data for Nepal is also minutely analyzed using the SIR model. Then, estimation is done for Kathmandu valley by regressing on the information gained from Nepal. The impact of government lockdown on the economic and mental health status is also estimated. It is on the basis of primary data. These data were collected from the online survey of 277 households.

The arrangement of this paper is in the following order. This section is followed by Materials and Methods. This is followed by Result and Discussion and Conclusion.

## 2. Materials and Methods

### 2.1. Data

This study is based on two sets of data. The first set of data is the daily COVID-19 updates published by the Ministry of Health and Population. Thus, it is a secondary data. It is published in the official website of Ministry of Health and Population, Government of Nepal, developed for COVID-19 updates [14]. It gives daily data of tested, infected, dead, recovered, active, and total infected, total recovered, and total dead cases. The data from 1 Feb 2021 to 1 June 2021 are taken for this study.

The second dataset is a primary data. An online survey of COVID-19 pandemic was conducted. A detailed structured questionnaire comprising 55 questions was designed. These questions were framed with the objective of knowing the impact of COVID-19 pandemic and the lockdown imposed on the economic, emotional, and psychological wellbeing of an individual. The sampling technique was snowball sampling. Here, the questionnaire was initially distributed to a set of university students. They were asked to provide the information about their families. They were further asked to collect information from five additional families. The importance of each question was explained to this initial set of respondents. They were requested to continue with this process with additional set of five people. This way the data were collected from 277 families comprising 1381 individuals. This large sample size ensured the statistical viability of the results obtained. This is by the application of Central Limit theorem. The data collection was done in two phases. One set of data was collected in November and December 2020. Another set was collected in March and April 2021. This survey collected information on the impact of lockdown imposed during the first wave of COVID-19 infection. The lockdown was imposed from 24 March 2020 to 19 September 2020.

The susceptible infected and recovered (SIR) time-dependent model for COVID-19 was as follows. Let *S*(*t*) represent the number of people susceptible to the disease on the day *t*. Let *X*(*t*) represent the number of infected from the disease on the day *t*. Let *R*(*t*) represent the number of people recovered from the disease on the day *t*. Here, *R*(*t*) means leaving the system either through recovery or through death on day *t*.

The following is assumed:

(1) $$\begin{array}{c} n\left(t\right)=S\left(t\right)+X\left(t\right)+R\left(t\right), \end{array}$$

were *n*(*t*) represents the number of people in the COVID-19 infection system on day *t*. It is assumed to be dependent on time. In the beginning of a pandemic, *n*(*t*) is a small number. As the pandemic spreads, this *n*(*t*) will be a larger number. At the end of the pandemic, this *n*(*t*) will shrink down to a smaller number. Let *β*(*t*) and *γ*(*t*) be the transmission rate and recovery rate of COVID-19 on day *t*.

The three variables *S*(*t*), *X*(*t*), and (*t*) are governed by the following differential equations [17, 18]:

(2) $$\begin{array}{c} \frac{dS\left(t\right)}{dt}=\frac{-\beta \left(t\right)S\left(t\right)X\left(t\right)}{n\left(t\right)}, \end{array}$$

(3) $$\begin{array}{c} \frac{dX\left(t\right)}{dt}=\frac{\beta \left(t\right)S\left(t\right)X\left(t\right)}{n\left(t\right)}-\gamma \left(t\right)X\left(t\right), \end{array}$$

(4) $$\begin{array}{c} \frac{dR\left(t\right)}{dt}=\gamma \left(t\right)X\left(t\right). \end{array}$$

The three variables *S*(*t*), *X*(*t*), and *R*(*t*) will satisfy (1).

Equations (2)–(4) are explained briefly in an intuitive way in the following manner. Here, (2) explains the difference in the number of susceptible on day *t*. Let us assume that the total people in the system of COVID-19 infection on day *t* are *n*(*t*). Then, the probability that a randomly chosen person is in the susceptible state is (*S*(*t*)/*n*(*t*)). Hence, an individual in the infected state will contact (*β*(*t*)*S*(*t*)/*n*(*t*)) people in the susceptible state per unit time. This implies that the number of newly infected is (*β*(*t*)*S*(*t*)*X*(*t*)/*n*(*t*)), as there are *X*(*t*) people in the infected state on day *t*. Thus, the number of people in the susceptible state on day *t* will decrease by (*β*(*t*)*S*(*t*)*X*(*t*)/*n*(*t*)). Further, every individual in the infected state will recover with rate *γ*(t); there are *γ*(*t*)*X*(*t*) recovered on day (*t*). This is shown in (4). This amount is subtracted in (3), to show the change in the number people of infected on day *t*.

Equations (2)–(4) are represented in discrete form in the following manner:

(5) $$\begin{array}{c} S\left(t+1\right)-S\left(t\right)=\frac{-\beta \left(t\right)S\left(t\right)X\left(t\right)}{n\left(t\right)}, \end{array}$$

(6) $$\begin{array}{c} X\left(t+1\right)-X\left(t\right)=\frac{\beta \left(t\right)S\left(t\right)X\left(t\right)}{n\left(t\right)}-\gamma \left(t\right)X\left(t\right), \end{array}$$

(7) $$\begin{array}{c} R\left(t+1\right)-R\left(t\right)=\gamma \left(t\right)X\left(t\right). \end{array}$$

So, from (6) and (7),

(8) $$\begin{array}{c} \beta \left(t\right)=\frac{X\left(t+1\right)-X\left(t\right)+R\left(t+1\right)-R\left(t\right)}{\left(S\left(t\right)X\left(t\right)/n\left(t\right)\right)}, \end{array}$$

(9) $$\begin{array}{c} \gamma \left(t\right)=\frac{R\left(t+1\right)-R\left(t\right)}{X\left(t\right)}. \end{array}$$

From (6)–(9), we get

(10) $$\begin{array}{c} \hat{X\left(t\right)}=\left[1-\hat{\gamma \left(t-1\right)}+\frac{\hat{\beta \left(t-1\right)}S\left(t-1\right)}{n\left(t-1\right)}\right]X\left(t-1\right). \end{array}$$

From (5)–(9), we get

(11) $$\begin{array}{c} \hat{S\left(t\right)}=S\left(t-1\right)-\frac{\hat{\beta \left(t-1\right)}S\left(t-1\right)I\left(t-1\right)}{n\left(t-1\right)}. \end{array}$$

From (7)–(9), we get

(12) $$\begin{array}{c} \hat{R\left(t\right)}=R\left(t-1\right)+\hat{\gamma \left(t-1\right)}I\left(t-1\right). \end{array}$$

### 2.2. Principal Component Analysis (PCA)

It is a statistical approach that can be used to analyze the interrelationship among a large number of variables [19]. Here, the information contained in a number of original variables is condensed into a smaller set of variates (factors). The loss of information is minimized in this process. This data summarization helps identify the underlying dimension or factor. It also estimates of factors and contribution of each variable to the factors (termed loadings). Unrotated factor matrix comprising of factor loadings is used when the main objective of research is in best linear combination of variables. Here, the particular combination of original variables account for more of variance in the data as a whole than any other linear combination.

Suppose we have a set of N variables, a^*∗*^1j to a^*∗*^Nj. These represent N variables related to the economic and mental health status with respect to COVID-19 lockdown, for each household *j*. Let us denote it as impact index. Further, let us standardize each variable by its mean and standard deviation; for example,  *a*_*ij*_=(*a*_1*j*_^*∗*^ − *a*_1_^*∗*^/*s*_1_^*∗*^), where *a*_1_^*∗*^  is the mean of *a*_1*j*_^*∗*^  across households and *s*_1_^*∗*^ is its standard deviation. These selected variables are expressed as linear combination of a set of underlying components for each household *j*:

(13) $$\begin{array}{c} a_{1j}^{*}=v_{11}*A_{1j}+v_{12}*A_{2j}+\ldots +v_{1N}*A_{Nj},\\ .\\ .\\ a_{Nj}^{*}=v_{N1}*A_{1j}+v_{N2}*A_{2j}+\ldots +v_{NN}*A_{Nj}, \end{array}$$

where j=1,   …,  J. A′s  are the components, and v′s are the coefficient on each component for each variable. The “scoring factors” from the model are recovered by inverting the system implied by (13) and yield a set of estimates for each of the *N* principal components:

(14) $$\begin{array}{c} A_{1j}=f_{11}*a_{1j}+f_{12}*a_{2j}+\cdots +f_{1N}*a_{Nj},\\ .\\ A_{Nj}=f_{N1}*a_{1j}+f_{N2}*a_{2j}+\cdots +f_{NN}*a_{Nj}, \end{array}$$

where *j*=1,…, *J*.

The impact index expressed in terms of the original (unnormalized) variables is therefore an index for each household based on the expression

(15) $$\begin{array}{c} A_{1j}=f_{11}\left(\frac{a_{1j}^{*}-a_{1}^{*}}{s_{1}^{*}}\right)+\cdots +f_{1N}\left(\frac{a_{Nj}^{*}-a_{N}^{*}}{s_{N}^{*}}\right), \end{array}$$

where *j*=1,   …, *J*.

With respect to the study of 277 households, there are 14 significant variables and 277 households. So, *N* = 14 and *J* = 277.

## 3. Result and Discussion

The results of this paper are given below in three parts. In the first, results from the detailed statistical analysis of data on COVID-19 pandemic survey are discussed. In the second part, the results from the time-dependent susceptible, infected, and recovered (SIR) model are given. In the third part, underreported infections and deaths in Kathmandu valley are estimated. It is for the period 1 Feb 2021 to 1 June 2021. In order to ensure continuity in the discussion of results, the research outcomes are ordered in the following manner.

**Figure 1:**
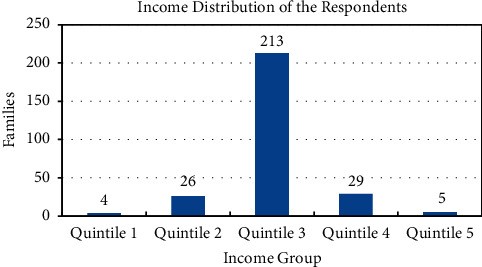
Income distribution of households.

**Figure 2:**
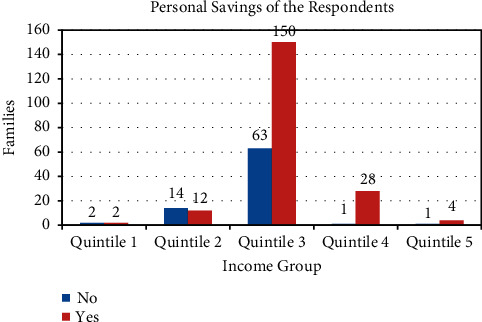
Personal savings of households.

**Figure 3:**
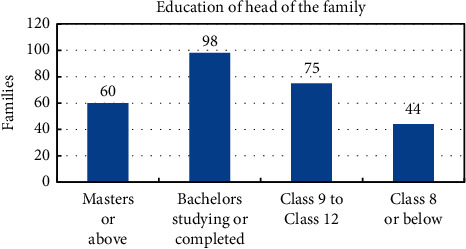
Educational qualification of the head of the family.

**Figure 4:**
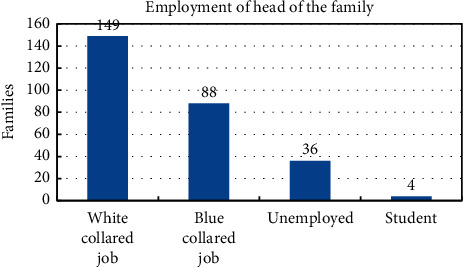
Type of employment of the head of the family.

A survey of 277 households was conducted. The responses were classified into categories for the categorical data analysis [20]. The aim was to assess the impact of lockdown on food security, economic status, and mental health of individuals. Information on 1371 individuals living in 277 households was gathered. The income group of these households classified into quintiles is shown in Figure 1. Here, the highest income group is represented by Quintile 5, and the middle income group is represented by Quintile 3. It is seen from Figure 1 that 213 households belong to middle income group, 5 belong to the highest income group, and 4 belong to the lowest income group.  Figure 2 shows the classification of these families according to their personal savings. We see that in Quintile 1 and Quintile 2, 50% or more of the families responded that they had no savings. But from Quintile 3 to Quintile 5, 50% or more responded that they had savings. The distribution of the families according to the educational qualifications and type of employment of the head of the family is given in Figures 3 and 4. We see that in 98 households, the head has studied till Bachelors level. This is followed by 75 households with educational background of Class 9 to Class 12. Only 60 households out of 277 households have highly educated head of the family. They have an education of Masters or above. Similarly, out of 277 households, in 149 households, the head of the family is employed in a white collared job. Information on the economic, educational, and professional status of these households is provided in Figure 1 to Figure 4.

The interdependence of income group with 31 variables related to food security, economy, and mental health was tested. Chi square test for independence of attributes was applied. The significant results indicating existence of dependence with the income group at 10% level of significance are shown in Table 1. So out of 31 variables, 14 variables were identified. These were dependent on the income status of the household. As seen from Table 1, these are related to food security, economy, and mental health status of the family during COVID-19 lockdown.

The descriptive statistics of these variables is given in Table 2. As seen from Table 2, these are categorical data that can be classified on ordinal scale. Out of 15 variables mentioned here, 13 range from 1 to 5. The response to personal savings is either yes or no. The response to fear of losing a job ranges from 0 to 5. The higher the value of these variables, the more severe is the negative impact of COVID-19 lockdown. The mean and SD values give the center of gravity and spread of the data.

The PCA of these 14 variables was conducted. These variables measure the impact of COVID-19 lockdown and are highly dependent on the income status. These 14 impact variables are correlated to one another. Through PCA, these 14 variables are reduced to 4 orthogonal uncorrelated variables explaining 58.998% of the total variance. The significance of this PCA is given in Table 3. The KMO is higher than 0.6. Bartlett test of sphericity rejects the null hypothesis. The null hypothesis here is that correlation matrix is an identity matrix. These results of Table 3 justify PCA for these data. The first principal component has high factor loadings on increased financial insecurity, decreased income, food shortage, depression, and anxiety. This component can be related to the overall negative impact of the lockdown. It accounts for 28.53% of the total variability. The second component has high loadings to limited variety and fewer foods available during this period. This component explains 14% of the total variability. The third component has high factor loading to financial insecurity and decreased income. It explains 8.709% of the total variability. The fourth component explains 7.676% of the total variability. It has high factor loadings on personal savings and fears of losing job.

**Figure 5:**
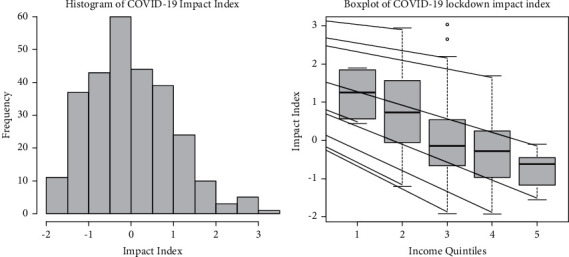
Behavior of impact index of COVID-19.

The dynamics of change in the impact of COVID-19 lockdown with change in income status and education was then studied. The COVID-19 lockdown impact index based on first principal component was regressed on household income and education of the head of the household. This COVID-19 impact index has mean = 0 and variance = 1. It ranges from -2 to 3.6. It quantifies the overall negative impact of lockdown. The higher the value of this index, the more severe the negative impact of this lockdown. Here, positive values indicate above average impact and negative values indicate below average impact. The significance of this regression is shown in Table 4. This linear regression is highly significant with a *p* value   ≤ 0.01. As seen from Table 4, the regressions coefficients are also highly significant with 5% level of significance. Although *R*^2^ is 0.1, regression and regression coefficients are highly significant. This helps us in making valid conclusions and generalizations. We see from Table 4 that with the increase in income status by 1 quintile, the negative impact of lockdown decreases by 0.477. Similarly, with 1 unit increase in education, the negative impact of lockdown decreases by 0.142. The inherent impact of COVID-19 lockdown is 1.811. This is a 96.4 percentile value. It lies in the top 3.5%. The behavior of COVID-19 impact index for 277 households is shown in histogram in Figure 5. The behavior of impact index, with respect to different income quintile over 277 households, is shown in the boxplot in Figure 5.

Here, the time-dependent SIR model was used. The dynamics of change in the daily number of infected was analyzed. The daily data on the number of people tested for COVID-19 were taken as data of daily number of susceptible cases. It was assumed that the number of tests performed per day was directly proportional to the number of susceptible per day. Fewer tests performed due to poor public health system meant underreported cases of susceptible and daily infections. Out of the total number of tests performed in a day, the number of tests with positive results indicated the number of those infected. Test with negative results indicated healthy people. Let *S*(*t*) represent the number of people tested for COVID-19 on day *t*. Here, *S*(*t*) is the number of susceptible on day *t*. Let *X*(*t*) denote the number of infected on day *t*. Let *D*(*t*) represent the number of dead on day *t*. Let *n*(*t*) represent the number of people in the system of COVID-19 infection on day *t*.

Here, *n*(*t*)=*S*(*t*)+*X*(*t*)+*R*(*t*)+*D*(*t*):

(16) $$\begin{array}{c} p_{X}\left(t\right)=\frac{X\left(t\right)}{S\left(t\right)}, \end{array}$$

(17) $$\begin{array}{c} p_{R}\left(t\right)=\frac{R\left(t\right)+D\left(t\right)}{S\left(t\right)}, \end{array}$$

(18) $$\begin{array}{c} p_{D}\left(t\right)=\frac{D\left(t\right)}{S\left(t\right)}, \end{array}$$

(19) $$\begin{array}{c} p_{S}\left(t\right)=\frac{S\left(t\right)}{n\left(t\right)}. \end{array}$$

Here a COVID-19 patient left a system when he/she recovered or died from the disease. A system is defined as the system of COVID-19 disease. The probability of an infected person is given by (16). The probability of a recovered person is given by (17). It is the probability of leaving the system. The probability of a dead person is given by (18). The probability of susceptible person in the COVID-19 system is given by (19).

**Figure 6:**
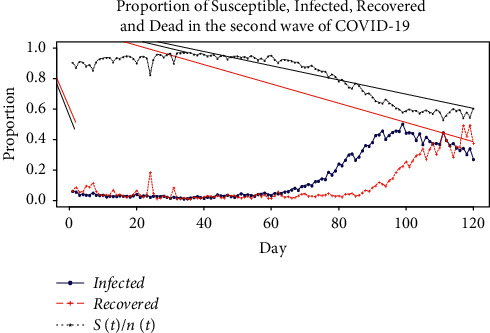
Behavior of proportion of infected, recovered, and susceptible COVID-19 patients for Nepal.

The behavior of *p*_*X*_(*t*), *p*_*R*_(*t*), and *p*_*S*_(*t*) is shown in Figure 6. This is observed over a period of 120 days. *p*_*X*_(*t*) is the probability of entering the system. *p*_*R*_(*t*) is the probability of leaving the system. We see from Figure 6 that the curve of proportion of those who were infected and recovered intersects at two points. The area between these two points of intersection indicates that a susceptible person is infected from COVID-19. But this person has not recovered from it. It is the probability that a susceptible person is in the system of COVID-19 infection. It is 0.692 from the data. So, if the entire population *x* is considered as susceptible in the peak of epidemic, then the number of those infected but not recovered is 0.692 x. Let us take Kathmandu valley as model area, and the population of Kathmandu valley is 1472000. So, in this second wave of COVID-19, 1018624 are infected. So next time, Nepal should prepare for 1018624 infected only from Kathmandu valley, in the entire wave of COVID-19 infection. Exiting from this system is either by recovery or death. This result is also validated on the primary data collected from the survey of 277 households comprising 1371 individuals. Here, 1371 individuals are taken as susceptible. They provided information of 885 COVID-19 infected. So, the proportion of those infected during the entire first wave was 0.641. This value is very close to the predicted proportion of COVID-19 during the second wave.

**Figure 7:**
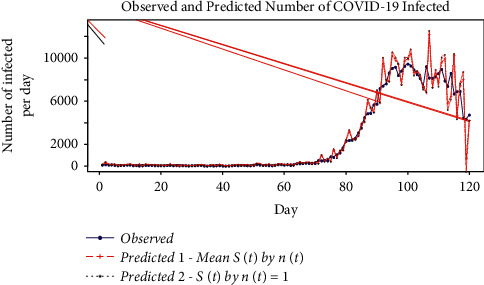
Observed versus predicted in the time-dependent SIR model for Nepal.

We also see from Figure 6 that (*S*(*t*)/*n*(*t*)) narrows down as the COVID-19 infected per day reaches its peak. This implies that the number of those susceptible tested positive for infection increases as the number of those infected increases. A comparison between observed and predicted number of infected people by fitting time-dependent SIR model is shown in Figure 4. Here, two models are compared. In Model 1, mean of (*S*(*t*)/*n*(*t*)) over all the values of *t* is considered. So, (*S*(*t*)/*n*(*t*))=mean(*S*(*t*)/*n*(*t*))=0.8403. Model 2 assumes that the number of susceptible at time *t* is equal to the total population at time *t*; that is, (*S*(*t*)/*n*(*t*)) ~ 1, ∀*t*. This is the general assumption in the case of the SIR model. The accuracy of these SIR models is given in Table 5. The accuracy of a model can be explained by *R*^2^, the coefficient of determination. As seen from Table 5, in the original time-dependent SIR model with (*S*(*t*)/*n*(*t*)), the *R*^2^ is 0.9571. This implies that 95.71% variability of the daily infected data for Nepal can be explained. *R*^2^ value of Model 1 is 0.930. Thus, this model can explain 93% variability of daily COVID-19 infected data. This is also seen by the close correspondence between observed and predicted value in Figure 7. In Model 2, *R*^2^ is 0.90. This model explains 90% variability of data. The higher *R*^2^, the closer fit between the observed and the predicted values. These models have shown very good results for the Kathmandu valley as well. In the original time-dependent SIR model with (*S*(*t*)/*n*(*t*)) , *R*^2^ is 0.968. This implies that 96.8% variability of daily infected data can be explained. As seen from Table 5, *R*^2^ for Model 1 is 0.956. *R*^2^ for Model 2 is 0.946. The results given in Table 5 validate and justify the assumptions made in the development of (16)–(19).

Kathmandu is the capital city of Nepal. Most of COVID-19 patients are found in Kathmandu valley. According to 2011 census, the population density of Nepal is 180. Kathmandu valley has the population density of 4416 [21]. Here, population density is defined as average number of people per square kilometer. Kathmandu valley comprises Kathmandu, Lalitpur, and Bhaktapur. It is an area with the highest population density in Nepal. The details of COVID-19 tests and testing facilities in Nepal are provided in Table 6. Most of the testing centers in Nepal are located in Kathmandu valley. It is located in Bagmati province of Nepal. Nepal has seven provinces. Out of 77 testing centers of Nepal, 42 are located in Bagmati province. Out of these, 34 testing centers are located in Kathmandu valley. It can be also seen from the table that the number of tests conducted and the number of positive cases are the highest in Kathmandu valley. In the beginning of the second wave on 1 Feb 2021, 4145 tests were conducted on one day in Nepal, out of which 3316 were only from Kathmandu valley. On this day, out of 159 positive cases across Nepal, 116 were from Kathmandu valley. It is also seen from the table that on 1 April 2021, 4213 tests were conducted in one day in Nepal. Out of these, 3204 were from Kathmandu valley. On this day, out of 152 positive cases from Nepal, 99 were from Kathmandu valley. There is a drastic increment in the number of test on 19 May 2021. Out of 21139 tests conducted in Nepal on this day, 9971 were from Kathmandu valley. Among 8064 positive cases in Nepal, 3101 are from Kathmandu valley. Out of 17147 tests conducted on 1 June 2021, 5886 were from Kathmandu valley. On this day, out of 5285 positive cases, 3065 were from Kathmandu valley.

We made following assumption here:

- The pattern exhibited by COVID-19 data for Nepal was correct but underreported.
- Kathmandu valley had the highest contribution to the daily numbers of COVID-19 susceptible, infected, recovered, and dead cases of Nepal. It was due to its highest population density.
- The data of tests on a day were taken as the data of number of susceptible on that day.
- The pattern exhibited by the daily numbers of COVID-19 susceptible, infected, recovered, and dead cases for Nepal was highly influenced by the data of these variables from Kathmandu valley.
- The highest weights were assigned to Kathmandu valley in the COVID-19 pandemic landscape of Nepal.

**Figure 8:**
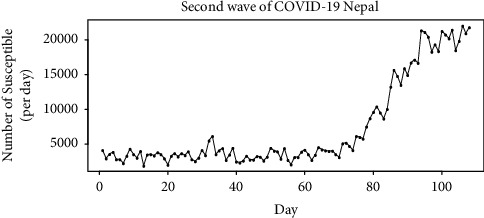
Number of susceptible cases per day from 1 Feb 2021.

This estimation is done in the following manner:

(i) The number of susceptible can be explained by the curve given in Figure 8. This can be explained by the parabolic function given in the following equation with *R*^2^=0.913:
  (20) $$\begin{array}{c} S\left(t\right)=3.840t^{2}-256t+6122. \end{array}$$
(ii) The daily number of susceptible cases for Kathmandu valley can be explained by the parabolic function [21].
These data are not available in the official data of COVID-19 published daily.
It is assumed that the behavior of the number of susceptible cases daily for Kathmandu valley is the same as that of Nepal.
So,
  (21) $$\begin{array}{c} S\left(t\right)=3.840t^{2}-256t+C. \end{array}$$
From COVID-19 updates, in 1 Feb 2021, 3316 tests were from Kathmandu valley. This can also be seen in Table 6.
Thus, *S*(1)=3316.
Then, from (6), we get
  (22) $$\begin{array}{c} C=S\left(1\right)-3.840+256=3316-3.840+256=3568.16. \end{array}$$
So,
  (23) $$\begin{array}{c} S\left(t\right)=3.840t^{2}-256t+3568.16. \end{array}$$
We can find predicted values of *S*(*t*) denoted by $\hat{S\left(t\right)}$ for *t*=1,2,…120 from (22). This is from 1 Feb 2021 to 1 June 2021. It is for a period of 120 days.
(iii) The probability of infection, death, and recovery in Kathmandu valley is assumed to be the same as that of Nepal. This is due to the highest concentration of COVID-19 patients here in comparison to any other part in Nepal. This can also be seen in Table 6.
Using (16) and (18) with $\hat{S\left(t\right)}$ obtained from (22), the daily number of infected $\hat{I\left(t\right)}$ and dead $\hat{D\left(t\right)}$ cases for Kathmandu valley can be estimated. The values of $\hat{S\left(t\right)}\text{ and }\hat{I\left(t\right)}$ are analyzed in detail using the SIR model given in *b*. The results of fitting of the SIR model on Kathmandu valley data are provided in Table 5.
(iv) The total underreported infections $=\sum_{t=1}^{120}\hat{\left(It\right)}$ − 113522 = 169511.7642 − 113522 = 55989.76.
The total underreported deaths $=\sum_{t=1}^{120}\hat{D\left(t\right)}$ − 1576  =  4326 − 1576 =2750.002.

COVID-19 daily updates for total infected and total dead in Kathmandu valley were used for the observed values of 113522 and 1576.

So, from 1 Feb to 1 June 2021, 2750.002 deaths and 55989 infections from COVID-19 are not reported.

These are reasonable estimates. The reasons behind these underreported cases are poor public health system and hence less testing, less contact tracing, and less infections reported. Some deaths took place at home and the causes could not be accounted for.

Various studies on underreporting of COVID-19 have been conducted. For example, Garcia et al. [22] used open online survey for indirect reporting of COVID-19. They compared the estimates obtained from the survey with serology study data of Spain. Krantz and Rao [23] used wavelet approach in harmonic analysis to develop full epidemic data from partial data. They used governmental data on COVID-19 for their analyses. Zhu et al. [24] developed wastewater SARS-CoV-2 RNA model based on fecal shedding profile of infected individuals. Rao and Krantz [25] commented on retrospective adjustment in the calculation of basic reproduction number, so that the reporting errors within the epidemic spread network could be correctly reported. Mackey et al. [26] used unsupervised learning approach. It was to detect and characterize user generated conversations in Twitter. These conversations were associated with COVID-19 related symptoms, experience with access to testing and mentions of disease recovery.

This paper is novel in comparison to the previously mentioned papers. Here, underreporting is studied from the perspective of a country with limited and scarce official records. Such countries have inefficient registration of vital events such as birth, death, and migration. Remote geographical locations, lack of awareness, and lack of incentives are some of the reasons behind this regrettable state. Keeping this backdrop in mind, the aim here was to develop a parsimonious model. This model should explain the scenario of COVID-19 accurately. This model should also have physical interpretation. The following steps were taken to fulfill these objectives:

1. Primary data of 277 households were collected from online survey.
2. The negative impact of COVID-19 lockdown was modeled using PCA.
3. The change in the governmental data of daily infected from COVID-19 was modeled using the time-dependent SIR model.
4. The accuracy of different time-dependent SIR models for Nepal and Kathmandu was measured.
5. The number of tests conducted per day in Kathmandu valley was assumed to be same as the number of susceptible cases per day. From partial data for Kathmandu valley, full data were generated using polynomial interpolation.
6. Underreported cases of infections and deaths were then predicted for Kathmandu valley.

## 4. Conclusion

In countries like Nepal, less testing, less contact tracing, and less reporting of COVID-19 have taken place. This is due to limited COVID-19 testing facilities. There are long queues in governmental testing facilities. Testing in private sector is expensive and beyond the reach of a common people.

Here, underreported cases of daily infections and daily deaths in Kathmandu valley are estimated. This is for a period from 1 Feb 2021 to 1 June 2021. Kathmandu valley is an area with the highest population density in Nepal. It comprises the capital city Kathmandu, Lalitpur, and Bhaktapur. Governmental records state that in Kathmandu valley, total COVID-19 infections and COVID-19 deaths during this time period are 113522 and 1576, respectively. But we predict 169512 and 4326, respectively. So, there are 55990 and 2750 cases of underreported infections and deaths, respectively. This was done under the assumption that patterns exhibited by governmental records were correct but underreported.

The time-dependent SIR model was also fitted to the COVID-19 data. Very high values of *R*^2^ for Nepal and Kathmandu were obtained. This validated and justified the assumptions made while using this SIR model. Under these assumptions, the probability that a person is in a second wave of COVID-19 infection is predicted to be 0.692. So, Kathmandu valley with a population of 1472000 should prepare for 1018624 infected in the next wave of COVID-19 infection.

COVID-19 lockdown was imposed from 24 March to 19 Sept 2020. Out of 31 variables related to economic and mental health status in this COVID-19 lockdown, 14 variables significantly related to the income status were identified. This was based on a primary data collected from an online survey of 277 households. Here, detailed structured questionnaire comprising 55 questions was designed. PCA was conducted on these 14 variables, and 4 principal components were identified. These components explained 58.998% variability of the data. The first principal component is related to the overall negative impact of the lockdown and explains 28.53% of the total variability. A COVID-19 lockdown impact index was calculated on the basis of the first principal component. Its behavior over 277 households is analyzed and displayed here with the help of histogram and boxplot. It has a mean value of 0 and standard deviation of 1 and ranges from −2 to 3.6. The higher the value of this COVID-19 impact index, the more severe the impact of the lockdown.

The regression of COVID-19 impact index on income status and educational background of the head of the family gave the following results. The inherent value of COVID-19 lockdown impact index is 1.811. This is a 96.4 percentile and top 3.6% value. As the income status increases by one quintile, the negative impact of COVID-19 lockdown decreases by 0.477. Similarly, with 1-unit increase in education, the negative impact of lockdown decreases by 0.142. These results are based on data collected from 277 households.

## Figures and Tables

**Table 1:** Results from chi square test of independence of attributes.

Sr. no.	Variable 1	Variable 2	Chi square	*p* value
1	Income group	Personal saving	17.983	0.0005
2		Worry about food during lockdown	27.154	0.02
3		Eat limited variety of food	20.8	0.093
4		Eat food that you did not want to eat	21.705	0.076
5		Eat fewer meals in a day	53.201	≤0.001
6		No food to eat of any kind in house	24.250	0.009
7		Increased food shortage	36.041	0.0015
8		Increase financial insecurity in house	25.876	0.028
9		Decreased income in my household	24.469	0.04
10		I had fears of losing the job	72.54	≤0.001
11		Depressed due to uncertainty created by COVID-19	22.717	0.0603
12		Anxious due to uncertainty created by COVID-19	24.704	0.037
13		News in media about COVID-19 increases my tension	24.6	0.0385
14		How have your stress and anxiety levels changed?	25.219	0.033

**Table 2:** Descriptive statistics of significant variables.

Sr. no.	Variables	Categorical data (ordinal)	Mean	SD
1.	Income group	Quintile 1 = 1, quintile 2 = 2, quintile 3 = 3, quintile 4 = 4, and quintile 5 = 5	3.018	0.573
2.	Personal savings	No = 1, Yes = 2	1.29	0.456
3.	Worry about food during lockdown	Never = 1, rarely = 2, sometimes = 3, often = 4, and always = 5	2.04	1.050
4.	Eat limited variety of food	Never = 1, occasionally = 2, sometimes = 3, often = 4, and always = 5	2.469	1.092
5.	Eat food that you did not want to eat	Never = 1, rarely = 2, sometimes = 3, often = 4, and always = 5	2.141	0.97
6.	Eat fewer meals in a day	Never = 1, rarely = 2, sometimes = 3, often = 4, and always = 5	1.708	0.891
7.	No food to eat of any kind in the house	Never = 1, rarely = 2, sometimes = 3, often = 4, and always = 5	1.224	0.571
8.	Increased food shortage	Never = 1, rarely = 2, sometimes = 3, often = 4, and always = 5	2.014	1.036
9.	Increased financial insecurity	Never = 1, rarely = 2, sometimes = 3, often = 4, and always = 5	2.668	1.176
10.	Decreased income in my household	Never = 1, rarely = 2, sometimes = 3, often = 4, always = 5	3.051	1.264
11.	I had fears of losing my job	Not applicable = 0, never = 1, rarely = 2, sometimes = 3, often = 4, and always = 5	1.744	1.366
12.	Depressed due to uncertainty of COVID-19	Never = 1, rarely = 2, sometimes = 3, often = 4, and always = 5	2.549	1.159
13.	Anxious due to uncertainty of COVID-19	Never = 1, rarely = 2, sometimes = 3, often = 4, and always = 5	2.805	1.138
14.	News of COVID-19 in media increased stress	Never = 1, rarely = 2, sometimes = 3, often = 4, and always = 5	3.516	1.075
15.	Change in stress level due to COVID-19	Never = 1, rarely = 2, sometimes = 3, often = 4, and always = 5	3.700	0.864

**Table 3:** Test for significance of PCA.

Kaiser–Meyer–Olkin measure of sampling adequacy	0.778
Bartlett's test of sphericity approx. chi square	1094
Df	91
Significance	0.000

**Table 4:** Results of linear regression of COVID-19 impact index on income status and education.

Model	Regression coefficients	*T*	Sig.
Intercept	1.811	5.628	.000
Household income	−0.477	−4.723	.000
Education of the head	−0.142	−2.428	.016

**Table 5:** Accuracy of SIR models in explaining daily number of COVID-19 infected cases.

Sr. no.	Region	Time-dependent SIR model	*R* ^2^
1	Nepal	(*S*(*t*)/*n*(*t*))	0.9571
2	Nepal	(*S*(*t*)/*n*(*t*))=mean(*S*(*t*)/*n*(*t*))=0.8403	0.930
3	Nepal	(*S*(*t*)/*n*(*t*))=1	0.90
5	Kathmandu	(*S*(*t*)/*n*(*t*))	0.968
6	Kathmandu	(*S*(*t*)/*n*(*t*))= mean(*S*(*t*)/*n*(*t*))=0.8577	0.956
7	Kathmandu	(*S*(*t*)/*n*(*t*))=1	0.946

**Table 6:** COVID-19 tests conducted in Nepal.

Regions	No. of testing centers	COVID-19 tests conducted in 24 hours							
		1 Feb		1 April		19 May		1 June	
		Tests	Positive	Tests	Positive	Tests	Positive	Tests	Positive
Province 1	9	135	4	408	1	2275	1208	2240	852
Province 2	8	31	1	44	4	1638	690	2058	424
Bagmati	42	3590	125	3249	100	10747	3491	7547	2738
Gandaki	3	123	22	180	24	1339	499	1085	394
Lumbini	9	253	7	300	20	2912	1027	2902	506
Karnali	2	13	0	0	0	684	352	495	206
Sudurpaschim	4	0	0	32	3	1544	797	820	165
Nepal	77	4145	159	4213	152	21139	8064	17147	5285
Kathmandu valley	34	3316	116	3204	99	9971	3101	5886	3065

Source: COVID-19 updates, Government of Nepal.

## Data Availability

The data used are described in the research article in detail.

## References

[1] Aljazeera (2020). Nepal to offer free COVID-19 tests and treatment as cases surge. https://www.aljazeera.com/news/2020/11/10/nepal-to-offer-free-covid-19-tests-and-treatment-as-cases-surge.

[2] Population Ministry of Health Nepal (2020). Health sector emergency response plan 2020. https://www.who.int/docs/default-source/nepal-documents/novel-coronavirus/health-sector-emergency-response-plan-covid-19-endorsed-may-2020.pdf?sfvrsn=ef831f44_2.

[3] Nepali Times (2021). 1^st^ sign of second wave in Nepal. https://www.nepalitimes.com/latest/first-signs-of-the-second-wave/.

[4] Baruah N., Arjal N. (2018). *Nepalese Labor Migration-A Status Report*.

[5] International Labour Organization (2020). Impact of COVID 19 on nepali migrant workers: protecting nepali migrant workers during health and economic Crisis 2020. https://www.ilo.org/wcmsp5/groups/public/---asia/---ro-bangkok/---ilo-kathmandu/documents/briefingnote/wcms_748917.pdf.

[6] Relief Web (2021). Thousands of jobless migrant workers take risky journey home to nepal. https://reliefweb.int/report/nepal/news-alert-thousands-jobless-migrant-workers-take-risky-journey-home-nepal.

[7] The Kathmandu Post (2020). Government preparing to bring stranded migrant workers home. https://kathmandupost.com/national/2020/05/21/government-preparing-to-bring-stranded-migrant-workers-home.

[8] The Kathmandu Post (2021). With hundreds of thousands of migrants predicted to return home, Nepal needs to brace for a crisis. https://kathmandupost.com/national/2020/04/22/with-hundreds-of-thousands-of-migrants-predicted-to-return-home-nepal-needs-to-brace-for-a-crisis.

[9] The Kathmandu Post (2020). Thousands of nepalese stranded in india lockdown urge their government to rescue them. https://kathmandupost.com/national/2020/04/01/thousands-of-nepalis-stranded-in-india-lockdown-urge-their-government-to-rescue-them.

[10] Garda World (2021). Nepal: officials extends COVID-19 control measuresin Kathmandu valley until at least June 3. https://www.garda.com/crisis24/news-alerts/483161/nepal-officials-extend-covid-19-control-measures-in-kathmandu-valley-until-at-least-june-3-update-45.

[11] The Record (2021). Coronavirus thriving in the Capital. https://www.recordnepal.com/coronavirus-thriving-in-the-capital.

[12] The Himalayan Times (2021). 3924 more contract COVID-19 in Kathmandu valley on Wednesday. https://thehimalayantimes.com/kathmandu/3924-more-contract-covid-19-in-kathmandu-valley-on-wednesday.

[13] Aljazeera As COVID wave rages in Nepal, hospitals run out of bed and oxygen. https://www.aljazeera.com/news/2021/5/21/nepal-struggles-to-cope-with-surging-covid-crisis.

[14] Ministry of Health and Population Nepal (2020). COVID 19 updates. https://covid19.mohp.gov.np/#/.

[15] Business Standard (2021). Coronavirus cases decline in Nepal, challenges remain with daily infections. https://www.business-standard.com/article/current-affairs/coronavirus-cases-decline-in-nepal-challenges-remain-with-daily-infections-121053100208_1.html.

[16] WHO (2021). Corona virus diseases 2021-country wise updates. https://covid19.who.int/region/searo/country/np.

[17] Almeshal A. M., Almazrouee A. I., Alenizi M. R., Alhajeri S. N. (2020). Forecasting the spread of COVID-19 in Kuwait using compartmental and logistic regression models. *Applied Sciences*.

[18] Chen (2021). A time dependent SIR model for COVID-19 with undetectable infected persons. *IEEE Transactions on Network Science and Engineering*.

[19] Bhuyan K. C. (2010). *Multivariate Analysis and its Applications*.

[20] Agresti A. (2007). *An Introduction to Categorical Data Analysis*.

[21] Statistics Central Bureau Nepal (2011). National population and housing census 2011 major highlights. https://cbs.gov.np/national-population-and-housing-census-2011-major-highlights/.

[22] Garcia A. A. (2021). Estimating the COVID-19 prevalence in Spain with indirect reporting via open surveys. *Frontiers in Public Health*.

[23] Kratz S. G., Rao A. S. R. S. (2020). Level of underreporting including under diagnosis before the first peak of COVID-19 in various countries: preliminary retrospective results based on wavelets and deterministic modeling. *Infection Control and Hospital Epidemiology*.

[24] Yifan Z. (2021). Early warning of COVID-19 via wastewater-based epidemiology: potential and bottlenecks. *The Science of the Total Environment*.

[25] Rao A. S. R. S., Krantz S. G. (2020). Ground reality versus model-based computation of basic reproductive numbers in epidemics. *Journal of Mathematical Analysis and Applications*.

[26] Mackey T., Purushothaman V., Li J. (2020). Machine learning to detect self-reporting of symptoms, testing access, and recovery associated with COVID-19 on Twitter: retrospective big data infoveillance study. *JMIR Public Health and Surveillance*.

